# Sperm Oxidative Stress Is Detrimental to Embryo Development: A Dose-Dependent Study Model and a New and More Sensitive Oxidative Status Evaluation

**DOI:** 10.1155/2016/8213071

**Published:** 2015-12-06

**Authors:** Letícia S. de Castro, Patrícia M. de Assis, Adriano F. P. Siqueira, Thais R. S. Hamilton, Camilla M. Mendes, João D. A. Losano, Marcílio Nichi, José A. Visintin, Mayra E. O. A. Assumpção

**Affiliations:** ^1^Laboratory of Spermatozoa Biology, Department of Animal Reproduction, School of Veterinary Medicine and Animal Science, University of Sao Paulo, Avenida Prof. Dr. Orlando Marques de Paiva 87, Cidade Universitária, 05508-270 Sao Paulo, SP, Brazil; ^2^Laboratory of In Vitro Fertilization, Cloning and Animal Transgenesis, Department of Animal Reproduction, School of Veterinary Medicine and Animal Science, University of Sao Paulo, Sao Paulo, Brazil; ^3^Laboratory of Andrology, Department of Animal Reproduction, School of Veterinary Medicine and Animal Science, University of Sao Paulo, Sao Paulo, Brazil

## Abstract

Our study aimed to assess the impact of sperm oxidative stress on embryo development by means of a dose-dependent model. In experiment 1, straws from five bulls were subjected to incubation with increasing H_2_O_2_ doses (0, 12.5, 25, and 50 *μ*M). Motility parameters were evaluated by Computed Assisted System Analysis (CASA). Experiment 2 was designed to study a high (50 *μ*M) and low dose (12.5 *μ*M) of H_2_O_2_ compared to a control (0 *μ*M). Samples were incubated and further used for* in vitro* fertilization. Analyses of motility (CASA), oxidative status (CellROX green and 2'-7' dichlorofluorescein diacetate), mitochondrial potential (JC-1), chromatin integrity (AO), and sperm capacitation status (chlortetracycline) were performed. Embryos were evaluated based on fast cleavage (30 h.p.i.), cleavage (*D* = 3), development (*D* = 5), and blastocyst rates (*D* = 8). We observed a dose-dependent deleterious effect of H_2_O_2_ on motility and increase on the percentages of positive cells for CellROX green, capacitated sperm, and AO. A decrease on cleavage and blastocyst rates was observed as H_2_O_2_ increased. Also, we detected a blockage on embryo development. We concluded that sperm when exposed to oxidative environment presents impaired motility traits, prooxidative status, and premature capacitation; such alterations resulting in embryo development fail.

## 1. Introduction

*In vitro* embryo production (IVP) in human represents an alternative for couples who are unable to naturally conceive, even after programmed intercourse or artificial insemination [1]. On the other hand, when focusing on animal reproduction, IVP is widely used with the main purpose of reducing the interval between generations, especially in cattle. In this scenario, Brazil stands out, responsible for 86% of* in vitro* produced embryos worldwide [2]. However, the extreme variability in IVP results limits the widespread use of this biotechnology.

One of the reasons for the inconsistent results of IVP is the individual effect of bull, known to strongly influence embryo development capacity [3, 4]. This may occur because spermatozoa may determine the moment [5] and the duration [6] of the first cleavage. In human, many studies have already demonstrated the influence of spermatozoa on embryo development, whether by extranuclear [7–9] or nuclear components [10–12].

*In vitro* and* in vivo* embryo production systems have some disparities with an important difference associated with oxygen concentrations. Values of approximately 20% of oxygen in the air normally used in IVP labs are superior to those found in the oviduct and uterus of most mammals [13]. The exposure of gametes and embryos to this excessive oxygen concentration during manipulations may lead to an inevitable increase in reactive oxygen species (ROS) production. A meta-analysis study in human has correlated increased ROS levels in the spermatozoa to subsequent impaired fertilization rate when using assisted reproduction techniques [14]. This result indicates that previous semen analysis for oxidative status may be essential towards attempts to predict IVP outcome and further course of procedures. In fact, previous study with primate oocytes undergoing intracytoplasmic sperm injection (ICSI) with spermatozoa exposed to oxidative stress revealed consequent fail in embryo development and high rates of blastomeric nuclear fragmentation [15]. Also, in bovine spermatozoa, Simões et al. [16] verified a negative correlation between sperm susceptibility to oxidative stress and cleavage and blastocyst rates. All these data suggest that spermatozoa when exposed to an oxidative environment may retain physical and chemical modifications potentially detrimental for embryo cytoplasmic and/or nuclear components, which may negatively affect embryo viability.

Another factor that may intensify sperm oxidative damage, influencing IVP results, is the process of cryopreservation, considering that the main source of male gametes for bovine* in vitro* fertilization is frozen semen. The process of cell cryopreservation has been related to ROS overproduction leading to cellular damage, especially due to lipid peroxidation, in different species including bovine sperm [17–20]. Also, during this process, the necessity of diluting or removing seminal plasma, the main source of antioxidant for spermatozoa, may increase the susceptibility of sperm to oxidative damage [21].

ROS generation in the spermatozoa can occur in the electron transport chain or though the NADPH oxidase activity [22]. Sperm energetic demand is extremely high and, therefore, mitochondrial activity is compensatively elevated. Probably, excessive mitochondrial ROS production may overcome the limited antioxidant machinery almost instantaneously. In sperm, ROS are known to participate in several physiological mechanisms such as capacitation, hyperactivation, and binding to the oocyte [23, 24]. Nevertheless, ROS are usually seen as a threat to cell integrity. Specific probes for ROS production show that free radicals may lead to membrane lipid peroxidation and decreased motility [25, 26]. Also, despite being highly compacted by protamine [27], sperm DNA is an important target for the attack of ROS, which leads to the formation of adducts between nitrogen bases, destabilizing the DNA molecule, resulting in DNA-strand breaks [28]. Studies indicate that even when DNA is damaged, sperm is still able to fertilize the oocyte; nonrepaired chromatin alterations have serious consequences for further embryo development [29–31]. In this context, our hypothesis is that bovine cryopreserved spermatozoa, when exposed to an oxidative environment, suffer injuries that will impact motility patterns and mitochondrial and DNA integrity, impairing fertilization ability and further* in vitro* embryo development. We evaluated the effect of oxidative stress induced by hydrogen peroxide on bovine sperm attributes (motility, mitochondrial membrane potential, oxidative and capacitation status, and DNA integrity) and subsequent* in vitro* embryo development. Furthermore, we propose a new and more sensitive flow cytometry method (CellROX green) to assess oxidative status of bovine sperm prior fertilization.

## 2. Material and Methods

This study was carried at the Animal Reproduction Department from the School of Veterinary Medicine and Animal Science of the University of São Paulo (VRA/FMVZ/USP). All procedures were performed according to the Bioethics Committee of the previously mentioned institution (protocol number 2710/2012).

### 2.1. Reagent and Solutions

All chemical reagents and solutions used in this study were purchased from Sigma-Aldrich (St. Louis, MO, USA) unless otherwise stated.

### 2.2. Experiment 1: Effect of Oxidative Stress Induction on Sperm Motility Related Variables

For this first experiment, thawed straws of the same batch from five Nelore bulls (*n* = 5), donated from Reproduction Centers, were used. Bulls were selected according to post-Percoll motility of at least 70%. This experiment was conducted in four replicates comprehended in a period of 2 weeks.

#### 2.2.1. Semen Processing

In order to maintain the same condition of semen processing prior to* in vitro* fertilization (IVF), experiment 1 was conducted as follows: each straw (0.25 mL) was thawed at 37°C for 30 seconds and subjected to Percoll gradient (45% and 90%) at 9000 G/5 minutes. Motile cells (pellet) were recovered and washed with 1 mL of Sp-TALP [32] at 9000 G/3 minutes. This final pellet was then resuspended to a final concentration of 25 × 10^6^ sptz/mL in Fert-TALP [32], with no capacitation agents (heparin, penicillamine, epinephrine, and hypotaurine). The same semen sample was divided between the experimental groups and incubated during 1 hour at 38.5°C, 5% CO_2_ in air, and high humidity.

#### 2.2.2. Oxidative Challenge with Hydrogen Peroxide

For oxidative stress induction, we used hydrogen peroxide 30% (Perhydrol, MERCK Millipore) diluted in Fert-TALP, for a final solution of 625 *μ*M. Hydrogen peroxide is not a free radical (i.e., one or more unpaired electrons); however, this peroxide is considered an important reactive oxygen species due to the high capacity to move across biological membranes and the high affinity with iron and copper ions to produce more unstable and reactive radicals such as the hydroxyl. Experimental groups were 0 (control), 12.5, 25, and 50 *μ*M of hydrogen peroxide.

#### 2.2.3. Computer Assisted Sperm Motility Analysis (CASA)

Motility parameters were evaluated using the Computer Assisted Sperm Analysis system (CASA; IVOS, v. 12.2, Hamilton Thorn Research, Beverly, MA). Settings used, previously described by Goovaerts et al. [33], were 30 frames at a frame rate of 60 frames/s; minimum contrast = 20; minimum cell size = 5 pixels; motility > 30 *μ*m/s; progressive motility > 50 *μ*m/s; straightness > 70%. In brief, each slide was heated at 37°C; 5 *μ*L of sample was placed in the slide and covered by a coverslip. A minimum of six fields were selected for analysis. Motility related variables considered were VAP (velocity average path), VCL (curvilinear velocity), VSL (straight-line velocity), BCF (beat cross frequency), ALH (amplitude of lateral head displacement), total and progressive motility, and percentage of cells with fast, medium, slow, and static movement. Among the previously mentioned variables, total and progressive motility were selected as more relevant for the selection of the hydrogen peroxide concentrations used in the second experiment.

### 2.3. Experiment 2: Effects of Sperm Oxidative Stress Challenge on Embryo Development

In this second experiment, for IVF, we used semen samples subjected to only two concentrations of hydrogen peroxide, selected according to experiment 1 results: 50 *μ*M (high concentration) and 12.5 *μ*M (low concentration) and a control group (0 *μ*M). This induction aimed to compare the impact of oxidative stress on cleavage, embryo development, and blastocyst rates. This experiment was conducted in 10 replicates, during two months, using 200 to 220 oocytes per replicate. For IVP control, semen samples with no H_2_O_2_ and not submitted to incubation were used and we considered only replicates with control blastocyst rate ≥20% (data not shown).

#### 2.3.1. *In Vitro* Embryo Production

Ovaries obtained from a slaughterhouse were transported to the laboratory in saline solution 0.9% at 30°C. Cumulus-oocytes complexes (COCs) were aspirated with an 18-gauge needle from 2 to 8 mm follicles. Oocytes with homogeneous ooplasm surrounded by more than two layers of compacted cumulus cells were selected for* in vitro* maturation (IVM). COCs selected were washed 3X in holding medium (TCM199 Hepes supplemented with 10% FCS (Gibco), 22 *μ*g/mL pyruvate, and 50 *μ*g/mL gentamycin) and 3X in IVM medium (TCM199 Bicarbonate supplemented with 10% FCS, 22 *μ*g/mL pyruvate, 50 *μ*g/mL gentamycin, 0.5 *μ*g/mL FSH Folltropin-V (Vetrepharm, Inc., Belleville, ON, Canada), 50 *μ*g/mL human chorionic gonadotrophin (Vetecor Laboratories, Calier, Spain), and 1 *μ*g/mL of 17*β*-estradiol) and placed for maturation in 90 *μ*L microdroplets of IVM medium (20–30 oocytes/drop), covered with mineral oil, during 22 to 24 hours at 38.5°C, 5% (v/v) CO_2_ in air, and high humidity.

For IVF, the same semen processing and induction protocol described in experiment 1 were performed (using only three concentrations of hydrogen peroxide, 0, 50, and 12.5 *μ*M). However, after concentration adjustment, we performed a pool of 3 bulls (same number of cells of each bull), in order to eliminate sire effect, and then divided the same sample between experimental groups. Matured oocytes were washed 3X in pre-IVF medium (TCM199 Hepes supplemented with 0.003% of BSA-V (m/v), 22 *μ*g/mL pyruvate, and 50 *μ*g/mL gentamycin) and 3X in Fert-TALP and placed for fertilization in 90 *μ*L microdroplets of Fert-TALP (20–30 oocytes/drop) covered with mineral oil. At the end of semen incubation, samples were washed with 550 *μ*L of Fert-TALP (9000 G/90 seconds). The sediment with sperm was recovered and used to inseminate microdroplets with oocytes (±100.000 sptz/drop) and the rest of the sample was used in subsequent sperm evaluations.

After IVF (18 hours; *D* = 1), putative zygotes were mechanically denuded by pipetting in pre-IVF medium and cultured in KSOM medium (Millipore Corporation, New Bedford, MA, USA) during 8 days at 38.5°C, 5% CO_2_, 5% O_2_, and 90% N_2_, under high humidity. On third day of culture (*D* = 3), KSOM was supplemented with FCS to a final drop concentration of 5%.

#### 2.3.2. Sperm Attributes Evaluations

CASA was performed as described in experiment 1. In addition, in this second experiment, we performed epifluorescence microscopy (Olympus IX80, Olympus Corporation, Tokyo, Japan) and flow cytometry evaluations (Guava EasyCyte Mini System, Guava Technologies, Hayward, CA, USA). This latter equipment contains a blue laser, which operates at 488 nm and emits a 20 mW visible laser radiation. A total of 10,000 events per sample were analyzed and data corresponding to yellow (PM1 photodetector, 583 nm), red (PM2 photodetector, 680 nm), and green fluorescent signals (PM3 photodetector, 525 nm) were recorded after a logarithmic amplification. For data analysis, cell doublets and debris were excluded using PM3/FSC (forward scatter) and all data was analyzed by FlowJo v10.2 software, except DNA integrity, which was evaluated using FlowJo v8.7 software.

#### 2.3.3. Mitochondrial Membrane Potential Evaluated by JC-1 Probe

Mitochondrial membrane potential was evaluated by JC-1 probe (5,5′,6,6′-tetrachloro-1,1′,3,3′-tetraethyl-benzimidazolylcarbocyanine chloride) (Invitrogen, Eugene, OR, USA). This probe emits green or red-orange fluorescence for low (LMM) or high mitochondrial potential (HMP), respectively. The procedure was performed with 187,500 cells diluted in Fert-TALP and stained with JC-1 (76.5 *μ*M in DMSO), in the dark at 37°C. Samples were analyzed by flow cytometry after 10 minutes, excited at 488 nm, and detected at 590 nm. For positive control, we used the protocol described by Celeghini et al. [34], with some modifications. A sample of semen submitted to 10 cycles of freezing and thawing in liquid nitrogen to disruption of membranes was used, and, for negative control, we used sperm sample processed as described in experiment 1.

#### 2.3.4. Oxidative Status Evaluated by 2′,7′-Dichlorofluorescein Diacetate (DCFH)

For this assay, 187,500 cells were stained with a solution containing DCFH and propidium iodide (PI) at a final concentration of 9.3 *μ*M and 6 *μ*M, respectively, in the dark at 37°C. Samples were analyzed by flow cytometry after 5 minutes, excited at 488 nm, and detected at 630–650 nm (PI) and 515–530 nm (DCFH). For data analysis, we selected the population of cells PI-DCFH+ (without membrane alteration and stressed).

#### 2.3.5. Oxidative Status Evaluated by CellROX Green

CellROX green (Molecular Probes, Eugene, OR, USA) is a fluorescent probe that penetrates the cell and, when oxidized by intracellular free radicals, binds to DNA, emitting a more intense green fluorescence. For this assay, 187,500 cells were stained with CellROX green (final concentration of 5 *μ*M) for 30 minutes at 37°C, and, in the last 10 minutes, PI was added to a final concentration of 6 *μ*M. Samples were analyzed by flow cytometry, excited at 488 nm, and detected at 630–650 nm (PI) and 515–530 nm (CellROX green). For data analysis, we selected the population of cells PI-VD+ (without membrane alteration and stressed). For CellROX green validation, we used increasing concentrations of hydrogen peroxide (0, 12.5, 50, and 200 *μ*M, during 1 hour at 38.5°C, 5% CO_2_, and high humidity), and the same sample stained with CellROX green was also stained with DCFH, in order to compare the two techniques.

#### 2.3.6. Chromatin Analysis

Chromatin stability assay was based on sperm chromatin structure assay (SCSA; [35], as described by Simões et al. [16]. This assay is based on an acid challenge that denatures DNA molecules from a susceptible chromatin structure, breaking hydrogen bounds and separating DNAs' strands, allowing acridine orange (AO) probe to intercalate and emit red (denatured single-strand DNA) or green (double-strand DNA) fluorescence. The procedure was performed with 375,000 cells. Samples were incubated with TNE buffer (Tris-HCl 0.01 M, NaCl 0.15 M, EDTA 1 mM, and distilled water, pH 7.4) and acid detergent (HCl 0.08 M, NaCl 0.15 M, and Triton X-100 0.1% in distilled water, pH 1.2). After 30 seconds, AO solution was added (citric acid 0.1 M, Na2HPO4 0.2 M, EDTA 0.001 M, NaCl 0.15 M, and AO stock 6 *μ*g/mL in distilled water, pH 6), and each sample was analyzed by flow cytometry after 5 minutes of incubation at 37°C, excited at 488 nm, and detected at 630–650 nm (red) and 515–530 nm (green). For positive control, a sample was incubated with hydrochloric acid (1.2 M in acid detergent, pH 0.1) and, for negative control, samples processed as described in experiment 1 were used.

#### 2.3.7. Capacitation Status Evaluated by Chlortetracycline Assay (CTC)

Capacitation status was evaluated by CTC assay as described by Ward and Storey [36] with some modifications. CTC penetrates through cellular membranes, increasing the fluorescent intensity when it binds with free calcium. An aliquot containing 375,000 cells was added to 20 *μ*L chlortetracycline solution, prepared in the same day of each replicate (CTC 38 *μ*M; stock solution: TRIS 20 mM, NaCl 130 mM, and L-cystein 4 mM). Samples were then fixed with 5 *μ*L paraformaldehyde 4%. An aliquot of this suspension was placed in a glass slide, mixed with DABCO solution (1,4-diazabicyclo[2.2.2]octane) and glycerol (1 : 9), covered with coverslip, and kept at −20°C, protected from light until the evaluation. Two hundred cells were examined, under epifluorescence microscope (Olympus IX80), using magnification of 1000x with mineral oil. Filters of 355 and 465 nm were used for excitation and emission, respectively. Three cellular categories were classified: noncapacitated (even distribution of yellow fluorescent over the head), capacitated (only acrosome region stained in yellow), and reacted (no yellow fluorescent in the head).

#### 2.3.8. Embryo Development Evaluations

Fast cleavage rate assessment was performed 30 hours after insemination (30 h.p.i), counting the number of structures that already showed fist cleavage at this point. Cleavage rate was assessed in the third day of culture (*D* = 3). Development rate was performed at fifth day of culture (*D* = 5), and, at this moment, embryos were classified in three categories according to developmental stage: noncleaved (NC), 2–4 cells, or 8–16 cells. Finally, at day eight of culture (*D* = 8), blastocyst rate was assessed. All these evaluations were made in stereomicroscope (Olympus TH3, Olympus Corporation) with 63x of magnification and the percentage of all rates was made over the total number of oocytes.

### 2.4. Statistical Analysis

Statistical analysis was performed using the software Statistical Analysis System 9.3 (SAS Institute, Cary, NC, USA). Data were tested for residue normality and variance homogeneity. Variables that did not comply with these statistical premises were subjected to transformations. We used PROC GLM for polynomial regression model in both experiments, considering treatment as main effect. On experiment 2, Spearman correlations analysis was performed to verify the correlation between variables analyzed, using PROC CORR procedure. In this case, groups were analyzed separately as control or treated (data of groups treated with 12.5 and 50 *μ*M of hydrogen peroxide were pooled). Results were reported as untransformed means ± SEM. All statistical analyses were calculated with a significance level of 5%.

## 3. Results

### 3.1. Experiment 1: Hydrogen Peroxide Promotes a Dose-Dependent Decrease in Sperm Motility Related Variables

**Figure 1:**
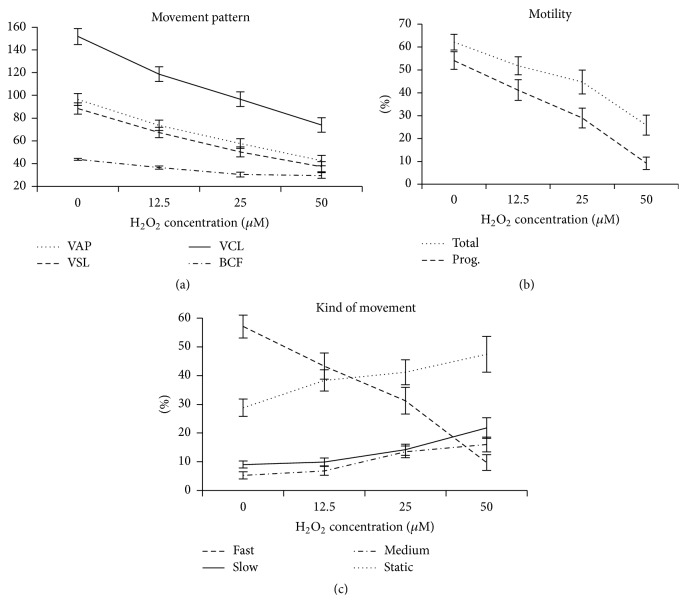
Spermatozoa motility parameters with treatment effect for hydrogen peroxide. (a) Units of VAP, VSL, and VCL = *μ*m/s and BCF = hertz; (b) Total: total motility and Prog.: progressive motility.

In the first experiment, we observed a negative effect of increasing concentrations of hydrogen peroxide for all motility related variables. This effect was evident for the velocity patterns revealed by the variables VAP, VSL, and VCL, which significantly decreased for all hydrogen peroxide concentrations. This decrease also occurred for BCF, however, in a more moderate pattern (Figure 1(a)). Total and progressive motility were also impaired while hydrogen peroxide concentrations increased, being more intensive between the concentrations of 25 and 50 *μ*M (Figure 1(b)). Similarly, for sperm populations with fast, medium, slow, and static movement, there was a decrease in sperm population with fast movement with a consequent increase in the remaining populations according to the increase in hydrogen peroxide concentrations (Figure 1(c)). Media values, straight-line equation, and *r* ^2^ values of all variables with treatment effect (*p* < 0.05) are present in Supplementary Material available online at http://dx.doi.org/10.1155/2016/8213071 (Table S1).

Based on the straight-line equation generated for total and progressive motility (Table S1), we chose, for experiment 2, 12.5 and 50 *μ*M concentrations of hydrogen peroxide. The criterion for such selection was a concentration that caused considerable oxidative damage but with acceptable motility for IVF (53% and 26% for total motility; and 41.7% and 8% for progressive motility, for the low and the high concentrations, resp.).

### 3.2. Experiment 2: Spermatozoa When Exposed to Hydrogen Peroxide Promote a Dose-Dependent Decrease in Embryo Development

#### 3.2.1. CellROX Green Validation

**Figure 2:**
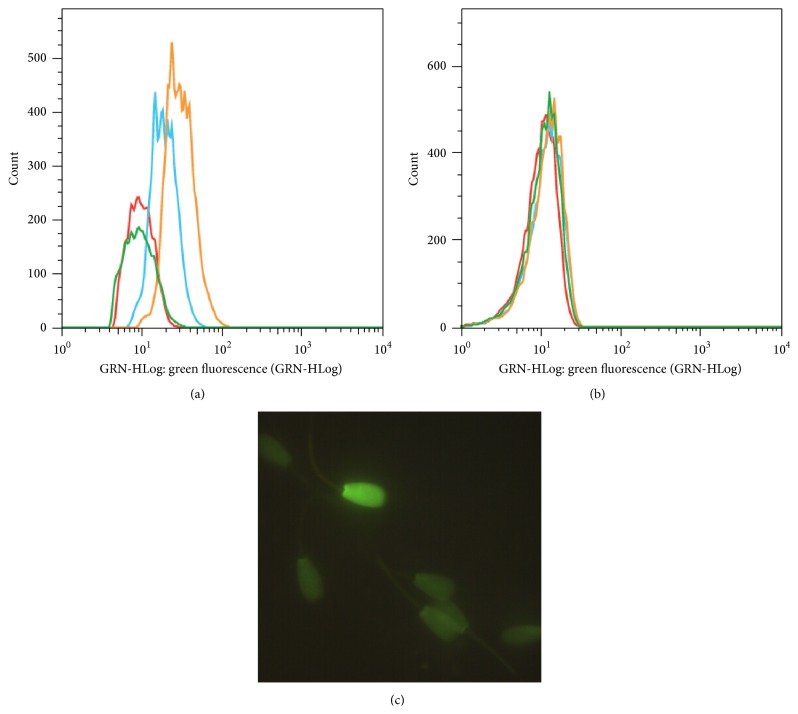
Histogram of green fluorescent intensity for CellROX green and DCFH probes and epifluorescence microscopy of spermatozoa stained with CellROX green. Histogram of green fluorescent intensity for CellROX green (a) and DCFH (b), wherein green lines correspond to control (without H_2_O_2_); red = 12,5 *μ*M H_2_O_2_; blue = 50 *μ*M H_2_O_2_; and orange = 200 *μ*M H_2_O_2_. Spermatozoa stained with CellROX green (c), positive (intense green) and negative cells (weak green), 1000x magnification with mineral oil.

In this validation, our results show more sensibility of CellROX green to detect increasing concentrations of hydrogen peroxide when compared to DCFH (Figure 2(a)). The same did not occur for DCFH (Figure 2(b)), since all concentrations have the same fluorescence intensity detected. We considered as more relevant, for both probes, the population of stressed cells with no membrane alteration (PI-DCFH+ or PI-VD+), once we speculate that these are the cells that still have the ability to fertilize the oocyte.

#### 3.2.2. Sperm Evaluations

**Figure 3:**
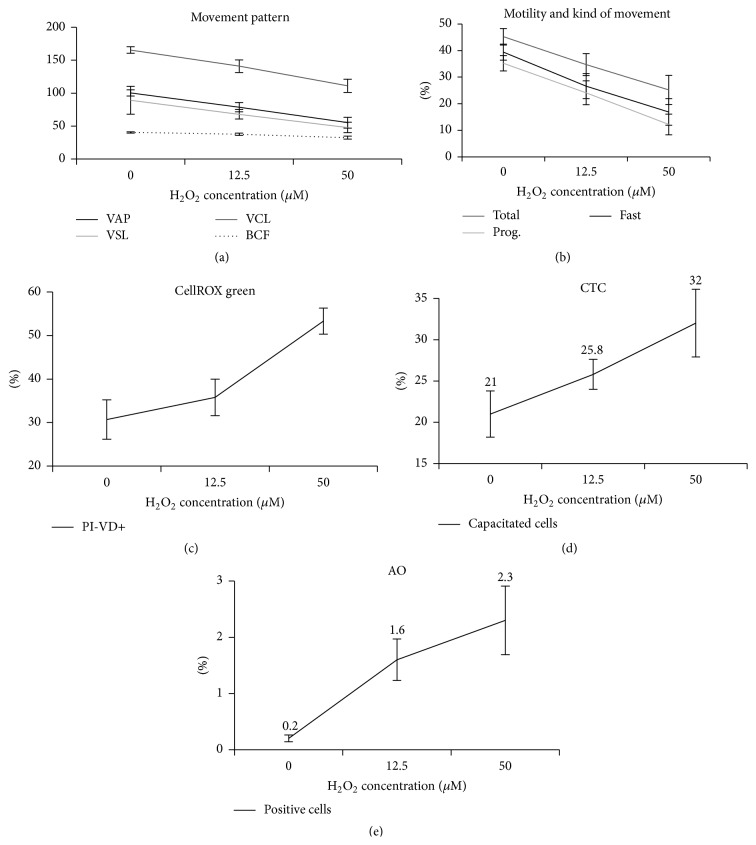
Spermatozoa evaluations with treatment effect for hydrogen peroxide. Units for VAP, VSL, and VCL = *μ*m/s and BCF = hertz (a); Total: total motility and Prog.: progressive motility (b); oxidative status evaluated by CellROX green represented by percentage of cells without membrane alterations and stressed (PI-VD+) (c); sperm capacitation evaluated by chlortetracycline assay (CTC) (d); and positive cells for AO (e).

The dose-dependent effect of oxidative stress on sperm motility occurred in the second experiment, similarly to the first experiment (Figures 3(a) and 3(b)). However, with additional sperm analyses, we observed this dose-dependent effect on oxidative and capacitation status, as evaluated by CellROX green and chlortetracycline assay, respectively. No treatment effect was observed for mitochondrial membrane potential (*p* = 0.13) nor oxidative status analyzed by DCFH (*p* = 0.09).

We observed an increase in the percentage of cells with no membrane alteration and stressed (PI-VD+; Figure 2(c)) and capacitated cells (Figure 3(d)) according to the increase of hydrogen peroxide concentrations. There was no effect of treatment for other categories related to capacitation status as evaluated by CTC assay (noncapacitated and reacted). Finally, Figure 3(e) shows the increase in the percentage of cells positive for AO (i.e., chromatin alteration), with increasing doses of hydrogen peroxide.

#### 3.2.3. Embryo Development

**Figure 4:**
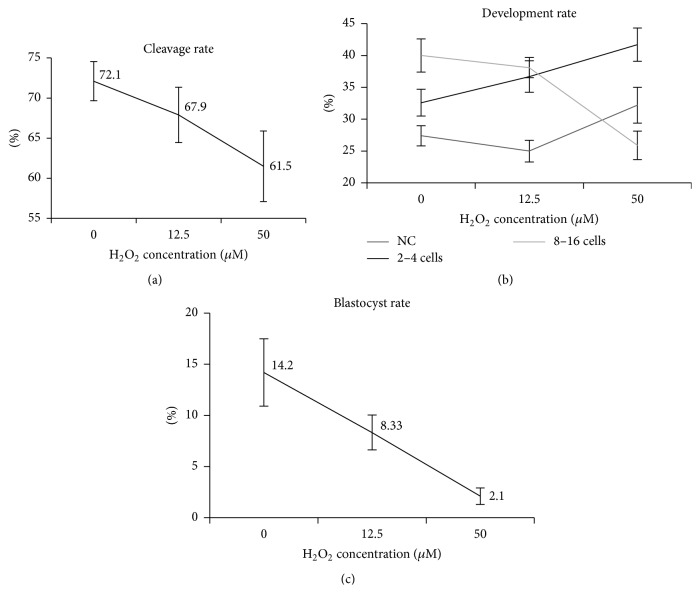
Embryo development evaluations. Cleavage rate evaluates on 3rd day of culture (a); development rate evaluates on 5th day of culture, wherein NC is noncleavage (b); and blastocyst rate evaluates on 8th day of culture (c).

For IVP, all parameters related to embryo development showed treatment effect, except for the fast cleavage rate (*p* = 0.15). Figures 4(a) and 4(c) show, respectively, the negative effect of hydrogen peroxide concentration on both cleavage and blastocyst rates.  Figure 4(b) illustrates embryo development rate, evaluated on the fifth day of culture. We observed an increase in the percentage of noncleaved (NC) and 2–4 cells embryos according to increase hydrogen peroxide concentrations. Contrarily, the number of 8–16 cells embryos decreased under the same hydrogen peroxide incubation protocol.

With these results, we verified that oxidative stress suffered by the sperm prior to IVF has a dose-dependent effect on both early (cleavage rate) and late (development rate and blastocyst) embryo development.

#### 3.2.4. Correlation between Sperm Parameters and Embryo Development

Correlation data are shown in Supplementary Material (Table S4). When sperm was treated with hydrogen peroxide, there was a positive correlation between the percentage of cells with high mitochondrial membrane potential and cells PI-VD+, and a negative correlation between the percentage of cells with high mitochondrial potential and movement pattern (VAP, VSL, VCL, and BCF).

For the oxidative status related variables, also in treated group, there was a negative correlation between stressed cells with no membrane alteration (PI-VD+ and PI-DCFH+) with movement pattern, and the percentage of cells PI-DCFH+ also correlates negatively with the total and progressive motility.

Finally, in the treated group, cleavage rate correlates positively with total and progressive motility, and blastocyst rate correlates negatively with the percentage of cells PI-VD+ and positively with velocity pattern (VAP, VSL, and VCL).

## 4. Discussion

In our study, we verified that sperm submitted to an oxidative environment negatively influence embryo development when used for* in vitro* fertilization. The severity of such effect is dependent on the intensity of the oxidative challenge. Interestingly, this impact can be observed not only on cleavage stage but also during the development to blastocyst. In addition, we verified the dose-dependent detrimental effect of oxidative stress on sperm motility, capacitation, and chromatin integrity. The regression model used in the preset work enables more inferences than studies which performed only mean comparisons. We can also consider the functioning of our treatment (hydrogen peroxide as oxidative stress promoter) over different biological sperm functions (motility, capacitation, embryo development, etc.), inferring over the effect of other doses not used in the present study, once the model generates a straight-line equation. Based on such equation, we may calculate the ideal hydrogen peroxide concentration for different desired effects (minimum effect on IVP, DNA damage, influence on motility, etc.). Also, for the first time, we reported the use of a new fluorescent probe, more efficient and sensible, to evaluate oxidative status by flow cytometry on bovine spermatozoa.

### 4.1. Oxidative Stress Impaired Sperm Motility, without Affecting Mitochondrial Membrane Potential

In experiment 1, we proposed a model to evaluate the effect of induced oxidative stress on bovine sperm motility patterns. Even though we used low concentrations of hydrogen peroxide in a short incubation period when compared to those previously used [37–39], our data suggests that bovine sperm motility is highly sensitive to oxidative stress induced by hydrogen peroxide. Furthermore, in treated group, the negative correlations between oxidative status (percentage of cells PI-VD+ and PI-DCFH+), sperm movement pattern (VAP, VSL, VCL, and BCF), and percentage of total and progressive motility reinforce the deleterious influence of the oxidative environment created by hydrogen peroxide on motility.

Hydrogen peroxide promotes a negative effect on motility patterns, but this effect apparently is not related to alteration on mitochondrial membrane potential. In boar sperm, hydrogen peroxide induces similar effect; reduction on sperm motility is not accompanied by impaired mitochondrial membrane potential or ATP concentrations [40]. These authors suggest that decreased motility caused by hydrogen peroxide is probably due to the action of the ROS in the contractility mechanisms of the sperm tail rather than impaired mitochondrial function. Another possibility is that the effect of hydrogen peroxide on mitochondrial membrane potential may be observed in a subsequent moment, as verified in other studies [20, 22, 26]. At this later stage, sperm is already dead, with impaired membrane integrity, and then the decreased mitochondrial membrane potential should be observed, similarly to cells submitted to the cryopreservation process [41].

The positive correlation between the percentage of sperm showing high mitochondrial membrane potential and those with signs of stress and no membrane alteration (PI-VD+) found in the treated group suggests that, in stressful conditions, intact and metabolically active cells would exhibit greater potential to release prooxidative substances. Similar relationship between mitochondrial membrane potential and ROS has been demonstrated in other cell types [42, 43]. Studies on heart and kidney cells hypothesize that mitochondrial membrane potential could be controlled by ATP/ADP production, since ATP synthesis is maximum in low membrane potential and ROS formation increases exponentially under high membrane potential (>140 mV) [44]. In fact, in our study, under the influence of hydrogen peroxide, samples showing higher percentages of sperm with high mitochondrial membrane potential presented impaired sperm movement pattern (VAP, VSL, VCL, and BCF). According to these results, we could speculate that, under stressful situations, intermediary to low mitochondrial membrane potential may be more favorable to motility, because in these conditions such cells would feature decreased potential to release intramitochondrial prooxidative factors.

### 4.2. Oxidative Stress and Sperm Capacitation

An important biological function of ROS on sperm physiology occurs in the capacitation process [45]. The action mechanism of hydrogen peroxide on sperm capacitation is still a matter of debate. However, previous studies demonstrated that, for bovine sperm, low concentrations of hydrogen peroxide (up to 50 *μ*M) promote a time- and dose-dependent effect on tyrosine phosphorylation of some proteins related to capacitation, with opposite effect under high concentrations (5 mM) [24]. In our study, oxidative stress induced by hydrogen peroxide also promoted a dose-dependent increase in the percentage of capacitated sperm. However, this increased number of capacitated sperm had no influence on sperm fertilization ability, evaluated indirectly by cleavage rate. Probably, under the challenge with hydrogen peroxide, such capacitation would occur prematurely, being actually an indication that these cells are dying, resulting in decreased sperm motility. Sperm cryopreservation, as well as successive washes, could anticipate the capacitation process by altering membrane's permeability and removing capacitation inhibition factors [46, 47]. In our study, hydrogen peroxide on higher concentrations probably exacerbated the premature capacitation, already initiated by the cryopreservation/thawing process, reducing the sperm limited lifespan and decreasing the ability to fertilize the oocyte. This is probably one of the reasons for the decreased cleavage rates on higher hydrogen peroxide concentrations.

### 4.3. CellROX Green Is More Efficient for Oxidative Status Evaluation on Bovine Spermatozoa

In our study, oxidative status was assessed by two fluorescent probes: 2′,7′-dichlorofluorescein diacetate (DCFH) and CellROX green. We observed differences in oxidative status detection between these two fluorescent probes. While, for DCFH, no dose-dependent effect for hydrogen peroxide was detected, for CellROX green, we could clearly observe an effect of increasing concentrations of hydrogen peroxide, indicating a difference of sensibility between these two probes to assess oxidative status.

To our knowledge, there is no previous report regarding the use of CellROX green in bovine sperm. A recent study validated CellROX deep red to detect oxidative status in ovine sperm [48], but in contrast to CellROX green that binds to DNA when it oxidizes, CellROX deep red detects cytoplasmic free radicals.

Both DCFH and CellROX green are considered nonspecific to the type of free radical detected, although some studies still use DCFH as a specific probe to detect hydrogen peroxide production [49]. In addition, these probes act in different cellular compartments. The signal generated by DCFH is most effective when the oxidizing agent is generated in the cytoplasm or near the plasma membrane [50]. On the other hand, CellROX green is considered primarily a nuclear probe. According to manufacture instructions, this probe has a weak basal fluorescence and, when oxidized, binds to DNA showing a bright and intense green fluorescence, which may also indicate a greater fluorescent stability.

The different sensibility of these two probes was observed in the validation for flow cytometry (Figure 2). When we used CellROX green, we could identify difference on green fluorescence intensity as hydrogen peroxide concentration increases. Such effect was not observed for DCFH; once it was independent of concentration, the green fluorescent was the same for all hydrogen peroxide concentrations. However, we must consider that samples used in the present study were frozen-thawed, subjected to many centrifugations (Percoll gradient and washes), and already highly susceptible to the oxidative stress [51]. Maybe this oxidative stress, inherent to this particular sample, was sufficient to generate positive signal for most of the cells evaluated using the DCFH. Also, some studies report the difficulty to work with this probe due to instability and photooxidation [52]. Under these experimental conditions, CellROX green proved to be more sensitive to and efficient in detecting oxidative status on bovine cryopreserved sperm cell submitted to an oxidative challenge.

### 4.4. Impact of Sperm Oxidative Status on* In Vitro* Embryo Development

In our experiment, we verified that sperm, when exposed to an oxidative environment, induce a dose-dependent effect on embryo development, from the first cleavage until blastocyst stage. The diminished cleavage rate probably occurred due to the lower percentage of motile cells as the concentration of the induction agent increased, which would then lead to impaired fertilization rates. In fact, the correlations found between motility and cleavage rate would agree with this hypothesis. In the control group, no correlation was found between motility and cleavage rate probably because, in this situation, sperm motility would remain high due to the Percoll gradient. However, in the treated group, there was a positive correlation between these two variables, suggesting that only under oxidative stressful conditions (the presence of hydrogen peroxide) sperm motility would strongly influence cleavage rates.

Embryo development rates evaluated on the fifth day of culture demonstrated that most embryos block the development at the 2–4 cells stage; at this moment, embryos should normally be at 8–16 cells stage. The influence of such developmental block would result in lower blastocyst rates. We can speculate two possible causes: (1) sperm that experienced an oxidative environment, after fertilization, may carry metabolites that would promote oocyte intracellular damage such as lipid peroxidation and antioxidant depletion, which would then impair initial embryo development (2–4 cells); (2) sperm DNA abnormalities induced by oxidative stress block embryo development even before embryo genome activation, leading to cellular division failure. The first hypothesis is very difficult to prove, once techniques currently available to assess zygotes' intracellular damage compromise further embryo development. Results of previous studies [29–31] give indications that sperm chromatin abnormalities are probably the most important reason for embryo development blockage.

In our study, we verified the effect of oxidative stress increasing the percentage of cells with chromatin alteration (AO+). Chromatin alterations in bovine sperm are relatively rare when compared to other species [16, 53, 54]. Despite the reduced percentage of cells positive for AO (2.3% for 50 *μ*M), hydrogen peroxide was capable of promoting a dose-dependent increase in the percentage of positive cells. Shaman and Ward [55] described that most of the DNA breaks, identified by the SCSA test, are located in the toroid linker regions. These chromatin regions can be more sensitive to oxidizing agents. As oxidation mechanism is dynamic, the DNA lesions started by hydrogen peroxide can be perpetuated within the cell even after fertilization, in these sensible regions and also on histone rich regions.

The retained histones on sperm DNA are not accidental. In an interesting study, Hammoud et al. [56] identified that retained histones are bound to developmental promoters, regions in* HOX* clusters, noncoding RNAs, and paternally expressed imprinted loci. This strategic localization of histones is considered important epigenetic markers of paternal DNA, indicating genes related to early embryo development that must be immediately activated after fertilization [57, 58]. However, the DNA attached to histones can be more susceptible to damage [55], exposing important epigenetic markers that, if damaged, may lead to embryo development arrest. Therefore, even under low percentage of cells with chromatin alteration, we can assume that the reason for embryo development block of our study is chromatin damage.

Embryo development fails due to spermatozoa exposed to oxidative stress which has been previously observed in primate [15]. In this study, authors attribute embryo development arrest before embryo genome activation to alterations during cellular division and high levels of nuclear fragmentation. In mouse, another study verified that spermatozoa exposed to hydrogen peroxide promote delay in embryo development and decrease in implantation rates [59]. Similar to our study, Simões et al. [16] identified lower cleavage rates in bovine sperm samples more susceptible to oxidative stress, but no effect was observed for blastocyst rate. Authors speculate that embryos that were able to surpass blocking phase are capable of reaching blastocyst stage. However, an increase in apoptotic blastomeres was observed. More studies directed to the early development period (between fist cleavage until 8–16 cells stage, when bovine embryo genome is activated) and epigenetic mechanisms may elucidate the dynamic of embryo development arrest caused by spermatozoa previously submitted to oxidative damage.

With the advances in transcriptome research, several studies have identified the presence of RNAs on spermatozoa [60–62]. Some of these RNAs have been reported as functional, necessary for important physiological events such as the first cleavage of zygotes [63]. RNA molecule is more unstable than DNA and also prone to damage due to ROS attack [64]. When oxidized, it can lead to formation of dysfunctional proteins, truncated or with incorrect folding [65]. In this context, we can speculate that some of these sperm RNAs can also be target of ROS oxidation, and once they are necessary for embryo development, this could be deleterious even before embryo genome activation.

## 5. Conclusion

We concluded that an oxidative environment can significantly impair bovine sperm motility pattern, oxidative and capacitation status, and DNA integrity. These changes would then reflect negatively on embryonic development from cleavage to blastocyst stage. Also, our study validated a new method to evaluate oxidative stress, CellROX green, which is more sensitive and efficient when compared to another assay normally used for sperm. More studies focusing on the moments between first cleavage and embryonic genome activation should be conducted aiming to better understand the deleterious effect of oxidized sperm in this particular period.

## Supplementary Material

Supplementary material provides statistic data information of sperm evaluations (experiment 1 and 2) and embryo development analysis not shown in the article.
