# Supplementary material for: Sperm Oxidative Stress Is Detrimental to Embryo Development: A Dose-Dependent Study Model and a New and More Sensitive Oxidative Status Evaluation
Source: Oxid Med Cell Longev. 2015 Dec 6;2016:8213071. doi: 10.1155/2016/8213071 (PMC4684862; doi:10.1155/2016/8213071)
Supplement: Supplementary file 1 — Supplementary material provides statistic data information of sperm evaluations (experiment 1 and 2) and embryo development analysis not shown in the article. [file 8213071.f1.docx]

Table S1 – Spermatozoa motility parameters. Mean and S.E.M., values of p, r^2^ and straight-linear equation for each variable analysis on experiment 1.

|  | 0 µM | 12.5 µM | 25 µM | 50 µM | p | r^2^ | Equation |
| --- | --- | --- | --- | --- | --- | --- | --- |
| VAP  (µm/s) | 96.3  (±5.2) | 73.6  (±4.6) | 57.6  (±4.2) | 42.7  (±4.5) | <0.01 | 0.89 | y = 96.2 – 2.03x + 0.01x^2^ |
| VSL  (µm/s) | 88.4  (±4.9) | 67.3  (±4.6) | 50.2  (±4.3) | 37.3  (±4.3) | <0.01 | 0.89 | y = 88.6 – 2x + 0.01x^2^ |
| VCL  (µm/s) | 151.7  (±6.9) | 118.6  (±6.4) | 96.6  (±6.4) | 73.9  (±6.3) | <0.01 | 0.89 | y = 4.9 – 0.02x + 0.0001x^2^ |
| BCF  (Hertz) | 43.8  (±0.8) | 36.5  (±1.4) | 30.4  (±2) | 29.6  (±2.4) | <0.01 | 0.82 | y = 1942.9 – 55.2x + 0.72x^2^ |
| Motility  (%) | 62.1  (±3.4) | 51.8  (±3.9) | 44.7  (±5.1) | 25.8  (±4.3) | <0.01 | 0.85 | y = 61.7 – 0.71x |
| Progressive  (%) | 54.1  (±3.8) | 41.2  (±4.4) | 28.9  (±4.3) | 9.2  (±2.6) | <0.01 | 0.88 | y = 52.8 – 0.89x |
| Fast  (%) | 57.0  (±3.9) | 43.3  (±4.5) | 31.2  (±4.6) | 9.7  (±2.7) | <0.01 | 0.88 | y = 55.8 – 0.93x |
| Medium  (%) | 5.2  (±1.2) | 6.8  (±1.5) | 13.4  (±2) | 16  (±2.5) | <0.01 | 0.80 | y = 5.4 + 0.22x |
| Slow  (%) | 9  (±1.2) | 9.9  (±1.3) | 14.2  (±1.9) | 21.7  (±3.5) | <0.01 | 0.83 | y = 1.91 + 0.02x |
| Static  (%) | 28.8  (±3) | 38.3  (±3.6) | 41.1  (±4.3) | 47.4  (±6.2) | 0.04 | 0.56 | y = 31.3 + 0.34x |

Legend: y = variable and x = H_2_O_2_ concentration

Table S2 – Spermatozoa motility parameters. Mean and S.E.M., values of p, r^2^ and straight-linear equation for each variable analysis on experiment 2.

|  | 0 µM | 12.5 µM | 50 µM | p | r^2^ | Equation |
| --- | --- | --- | --- | --- | --- | --- |
| VAP  (µm/s) | 100.3 (±4.8) | 78.7 (±6.9) | 55.4 (±8.2) | < 0.01 | 0.73 | y = 95.5 – 0.83x |
| VSL  (µm/s) | 89.3 (±21.1) | 68 (±7.1) | 48 (±8) | <0.01 | 0.72 | y = 84.2 – 0.75x |
| VCL  (µm/s) | 165.4 (±4.9) | 140.9 (±9.4) | 111.2 (±10.1) | <0.01 | 0.75 | y = 160.3 – 1.02x |
| BCF  (Hertz) | 40.5 (0.8) | 37.6 (±1.8) | 32.6 (±2.2) | <0.01 | 0.56 | y = 66507.3 – 518.4x |
| Motility  (%) | 45.2 (±3.1) | 34.7 (±4.1) | 25.2 (±5.4) | <0.01 | 0.65 | y = 42.7 – 0.36x |
| Progressive  (%) | 35.2 (±2.8) | 24.1 (±4.5) | 12.2 (±3.9) | <0.01 | 0.65 | y = 32.7 – 0.42x |
| Fast  (%) | 39.4 (±3) | 26.6 (±4.7) | 16.9 (±5) | <0.01 | 0.65 | y = 36.1 – 0.40x |
| Capacitated  (%) | 21 (±2.8) | 25.8 (±1.8) | 32 (±4.1) | 0.018 | 0.78 | y = 21.97 + 0.21x |
| PI-VD+  (%) | 30.7 (±4.5) | 35.8 (±4.2) | 53.3 (±3) | <0.01 | 0.91 | y = 1135.7 + 35.8x |
| AO+  (%) | 0.2 (±0.06) | 1.6 (±0.3) | 2.3 (±0.6) | <0.01 | 0.89 | y = 1.19 – 0.04x |

Legend: y = variable and x = H_2_O_2_ concentration

Table S3 – Embryo development evaluations. Mean and S.E.M., values of p, r^2^ and straight-linear equation for each variable analysis

|  | 0 µM | 12.5 µM | 50 µM | p | r^2^ | Equation |
| --- | --- | --- | --- | --- | --- | --- |
| Cleavage rate  (%) | 72.1 (±2.4) | 67.9 (±3.4) | 61.5 (±4.4) | 0.02 | 0.71 | y = 71.4 – 0.2x |
| Non-cleavage  (%) | 27.4 (±1.6) | 25 (±1.7) | 32.2 (±2.8) | 0.04 | 0.63 | y = 25.7 + 0.11x |
| 2-4 cells  (%) | 32.6 (±2.1) | 36.7 (±2.4) | 41.7 (±2.6) | <0.01 | 0.75 | y = 33.4 + 0.17x |
| 8-16 cells  (%) | 40 (±2.6) | 38.1 (±1.6) | 25.9 (±2.2) | <0.01 | 0.76 | y = 40.7 – 0.29x |
| Blastocyst rate  (%) | 14.2 (±3.3) | 8.33 (±1.7) | 2.1 (±0.8) | 0.01 | 0.57 | y = 2.4 – 0.02x |

Legend: y = variable and x = H_2_O_2_ concentration. Cleavage rate (D=3); Non-cleavage, 2-4 cells and 8-16 cells (D=5) and blastocyst rate (D=8).

Table S4 – Correlations between spermatozoa evaluations and embryo development of experiment 2 for control (without H_2_O_2_) and treated (with H_2_O_2_)

|  | **Cap** | **PI-VD+** | **PI-**  **DCFH+** | **APM** | **VAP** | **VSL** | **VCL** | **ALH** | **BCF** | **Motil** | **Prog** | **AO+** | **Cliv** | **Blast** |
| --- | --- | --- | --- | --- | --- | --- | --- | --- | --- | --- | --- | --- | --- | --- |
| **Cap** |  | ns | ns | ns | ns | ns | ns | -0.58^a^ | ns | ns | ns | ns | ns | ns |
| **PI-**  **VD+** | -0.83^a^ |  | ns | 0.64^b^ | -0.49^a^ | -0.43^a^ | -0.55^b^ | ns | 0.42^a^ | ns | ns | ns | ns | -0.49^a^ |
| **PI-**  **DCFH+** | ns | ns |  | ns | -0.42^a^ | -0.47^b^ | ns | ns | -0.43^a^ | -0.62^b^ | -0.6^b^ | ns | ns | ns |
| **APM** | ns | ns | ns |  | -0.53^b^ | -0.49^b^ | -0.50^b^ | ns | -0.39^a^ | ns | ns | ns | ns | ns |
| **VAP** | ns | ns | ns | ns |  | 0.99^b^ | 0.90^b^ | ns | 0.77^b^ | 0.67^b^ | 0.83^b^ | ns | ns | 0.52^a^ |
| **VSL** | ns | ns | ns | ns | 0.98^b^ |  | 0.86^b^ | ns | 0.78^b^ | 0.68^b^ | 0.84^b^ | ns | ns | 0.53^a^ |
| **VCL** | ns | ns | ns | ns | ns | ns |  | 0.42^a^ | 0.76^b^ | 0.65^b^ | 0.74^b^ | ns | ns | 0.56^a^ |
| **ALH** | ns | ns | ns | ns | ns | ns | 0.61^a^ |  | ns | 0.39^a^ | ns | ns | ns | ns |
| **BCF** | ns | ns | ns | ns | ns | ns | ns | ns |  | 0.57^a^ | 0.61^b^ | ns | ns | 0.51^a^ |
| **Motil** | ns | ns | ns | ns | ns | ns | ns | ns | ns |  | 0.93^b^ | ns | 0.55^b^ | ns |
| **Prog** | ns | ns | ns | ns | ns | 0.55^a^ | ns | ns | ns | 0.95^b^ |  | ns | 0.44^a^ | ns |
| **AO+** | ns | ns | ns | ns | ns | ns | ns | ns | ns | ns | ns |  | ns | ns |
| **Cliv** | ns | ns | -0.73^b^ | ns | -0.77^b^ | -0.78^b^ | ns | ns | ns | ns | ns | 0.7^a^ |  | ns |
| **Blast** | ns | ns | ns | ns | ns | ns | ns | ns | ns | ns | ns | ns | ns |  |

Legend: ^a^p<0.05 ^b^p<0.01. Gray cell = control (without H_2_O_2_) and White cells = treated (with H_2_O_2_). Cap = % capacitated; APM = % high mitochondrial potential; VAP, VSL and VCL = µm/s; BCF = hertz; ALH = µm; Motil = % total motility; Prog = % progressive motility; Cliv = cleavage rate (%); Blast = blastocyst rate (%).
